# Vaginal microbiome composition in women with HIV undergoing treatment of cervical transformation zone in a screen and treat program in Zambia

**DOI:** 10.1097/QAD.0000000000004187

**Published:** 2025-06-26

**Authors:** Leeya F. Pinder, Pierre Bertrand, Veronica Fertitta, Vincent Cahais, Mulindi Mwanahamuntu, Namakau Nyambe, Samson Chisele, Aaron Lunda Shibemba, Sandrine McKay-Chopin, Cyrille Cuenin, Eric Lucas, Michael Korenjak, Richard Muwonge, Akram Ghantous, Zdenko Herceg, Groesbeck P. Parham, Jiri Zavadil, Partha Basu, Tarik Gheit

**Affiliations:** a Department of Obstetrics and Gynecology, Women and Newborn Hospital, Lusaka, Zambia; b Division of Gynecologic Oncology, University of Washington, Seattle, Washington, USA; c Epigenomics and Mechanisms Branch, International Agency for Research on Cancer, Lyon, France; d University Teaching Hospital; e University of Zambia School of Medicine, Lusaka, Zambia; f Early Detection, Prevention & Infections Branch, International Agency for Research on Cancer, Lyon, France.

## Abstract

This study assessed the vaginal microbiome in women with HIV undergoing cervical transformation zone treatment in Zambia. 16S rRNA sequencing showed lower microbial diversity in successful outcomes (*N* = 18) than those with treatment failure (*N* = 17) treatment outcome, with *Lactobacillus* abundance correlated with success. Moreover, HPV-negative women (*N* = 12) had higher *Lactobacillus* levels and less pathogens than HPV-positive women (*N* = 12). These findings suggest a *Lactobacillus*-dominated microbiome may be associated with positive treatment outcomes.

In several low and middle-income countries (LMICs), cervical cancer represents the most common cancer among women and can constitute up to a quarter of all female cancers [1]. In 2018, Zambia ranked third globally in cervical cancer incidence, reporting 66.4 new cases per 100 000 women and 1839 deaths annually [2–6]. A significant proportion of cervical cancers in LMICs, especially in sub-Saharan Africa, are attributed to a high burden of HIV [7–10]. While the immune system typically clears most human papillomavirus (HPV) infections and resolves precancerous lesions spontaneously, relatively little is known about the factors that facilitate oncogenic HPV persistence and progression to neoplasia [11], especially among women with HIV (WWH).

Several studies have reported that a reduced abundance of *Lactobacillus* spp. combined with a high microbiome diversity is associated with an increased risk of HPV acquisition, HPV persistence, and progression to high-grade cervical intraepithelial neoplasia (CIN) and cancer (Reviewed in: [12–15]).

Recent findings have shown that treating cervical precancer in WWH is significantly less effective than in women without HIV [16–19]. A screen-and-treat study in Zambia involving 750 women positive on VIA (visual inspection with acetic acid) found that treatment success rates were much lower in WWH across different treatment methods [18]. The trial subsequently recruited additional 2373 women, and the difference in treatment outcome between WWH and women without HIV remained significant [20], thus raising questions about a potential role of the microbiome.

In this study, we report the evolution of the vaginal microbiome evaluated by 16S rRNA sequencing and compared the microbiome composition between the outcomes of treatment of cervical transformation zone, in VIA-positive WWH at baseline. Within the framework of the randomized controlled trial conducted at cervical screening clinics in Zambia [20], cervicovaginal cells were gathered from 46 WWH undergoing treatment. Vaginal cells were collected at three time points: immediately prior to treatment (baseline), and at 3 and 12 months posttreatment. This study received approval from the research ethics committees at International Agency for Research on Cancer (IARC; France), the University of North Carolina, the University of Zambia, and the National Health Research Agency of Zambia. Every participant provided written informed consent.

Sequencing libraries were prepared following the Illumina protocol “16S Metagenomic Sequencing Library Preparation” for the Illumina MiSeq System (https://support.illumina.com/documents/documentation/chemistry_documentation/16s/16s-metagenomic-library-prep-guide-15044223-b.pdf). The sequencing data were analyzed using a publicly available bioinformatics pipeline (https://github.com/IARCbioinfo/wsearch-nf).

Treatment efficacy was assessed at 12 months as follows: **Treatment failure** was defined as HPV persistence during follow-up with the same HPV type detected at baseline or as a persistent VIA lesion during follow-up among women who were HPV-negative at baseline. **Treatment success** was defined as HPV clearance during follow-up of the same HPV type detected at baseline or a VIA-negative finding at follow-up among women who were HPV-negative at baseline.

Participants with an unknown follow-up outcome status were those for whom no follow-up information was available. Of note, follow-up VIA findings were used among women with baseline HPV-negative results since follow-up HPV testing was done only among women with baseline HPV-positive results as was stated in the protocol.

Among the 46 WWH, treatment outcomes at 12 months were available for 35 participants. Of these, 17 experienced treatment failure while 18 had successful outcomes (Supplementary Table S1). Of the 138 expected vaginal samples, 123 were available for microbiome analysis across the three time points, but 25 samples were excluded due to poor sequencing quality. Potential causes for this include DNA quality issues (e.g., degraded DNA) or unexpected errors during library preparation and sequencing.

First, an analysis of the alpha diversity using the standard Shannon index was performed to measure both species richness and evenness at baseline, 3 months, and 12 months. The alpha diversity remained stable at baseline and at 3 months with a Shannon index median of 2.58 [IQR, 2.13–2.89] and 2.60 [IQR, 2.01–2.99], respectively, then slightly decreased at 12 months (2.44 [IQR, 1.16–2.89]) (Supplementary Fig. 1). No significant difference was found between paired women (*N* = 25) at baseline and 12 months (*P* > 0.05) (Fig. 1a). When comparing alpha diversity between treatment outcomes, paired women who experienced treatment success (*N* = 13) showed a median alpha diversity value of 2.30 at baseline and 2.22 at 12 months, while those who experienced treatment failure (*N* = 10) had values of 2.54 at both time points. There was no statistically significant difference between “Success” and “Failure” groups at 12 months (*P* > 0.05) (Fig. 1b).

**Fig. 1:**
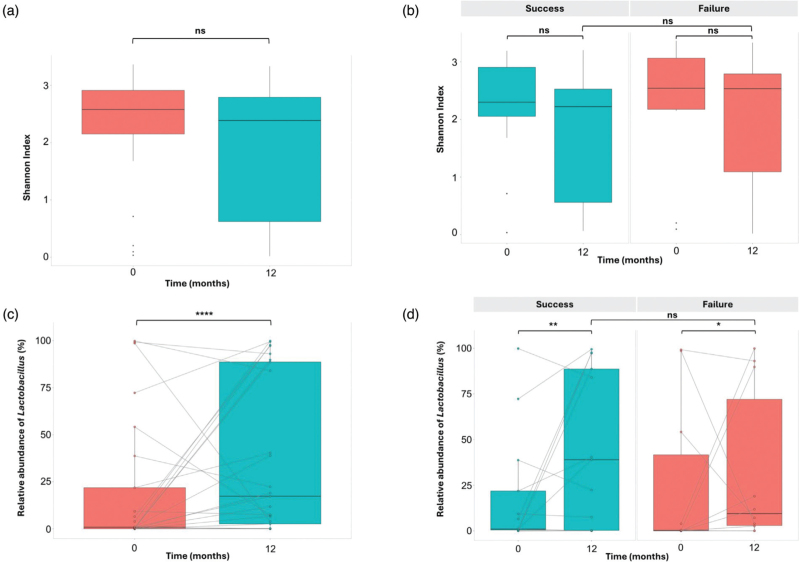
Analysis of alpha diversity and Lactobacillus relative abundance.

(a) Alpha diversity at baseline and 12 months, irrespective of treatment outcome (Wilcoxon signed-rank test, not significant [ns], *P* > 0.05). (b) Alpha diversity at baseline and 12 months by treatment outcome. Wilcoxon signed-rank test: ns, *P* > 0.05; Mann--Whitney test: ns, *P* > 0.05. (c) Relative abundance (%) of *Lactobacillus* at baseline and 12 months, regardless of treatment outcome (Wilcoxon signed-rank test, ^∗∗∗∗^*P* < 0.0001). (d) Relative abundance (%) of *Lactobacillus* stratified by treatment outcome. Wilcoxon signed-rank test: ^∗∗^*P* < 0.01, ^∗^*P* < 0.05; Mann--Whitney test: ns, *P* > 0.05.

The analysis of the beta diversity analysis also showed no significant difference between both groups (PERMANOVA, *P* > 0.05) (Supplementary Fig. 2).

However, notable trends in microbiome composition emerged (Supplementary Fig. 3). In the treatment success group, the relative abundance of *Lactobacillus* increased significantly over time, from 19.3% at baseline, 30.5% at 3 months, and 37.4% at 12 months (Cochran–Armitage test, Z = 213.41, *P* < 0.05). Concurrently, the abundance of potential pathogens such as *Prevotella* (12.7, 11.5, and 9.2%), *Shuttleworthia* (10.4, 1.7, and 5.9%), *Fastidiosipila* (3.3, 2.2, and 1.8%), and *Sneathia* (10.7, 14.0, and 7.0%) decreased over time. In contrast, the treatment failure group exhibited a slower increase in *Lactobacillus* (19.1, 24.0, and 30.7%), although the increasing trend was significant (Z = 135.74, *P* < 0.05), and a stable relative abundance of the pathogens mentioned above (32.1, 26.8, and 27.8%). Notably*, Porphyromonas* persisted at 3 and 12 months in the treatment failure group (4.7 and 2.0%, respectively) but was absent in the treatment success group.

Next, we focused the microbiome composition analysis on paired women only. Regardless of treatment outcome, the median relative abundance of *Lactobacillus* differed significantly between baseline and 12 months (0.88 vs. 17.32%, *P* < 0.0001) in paired women (*N* = 25) (Fig. 1c). In the treatment success group (*N* = 13), the median relative abundance of *Lactobacillus* increased significantly from baseline to 12 months (1.00 vs. 38.97%, *P* < 0.01). In the treatment failure group (*N* = 10), a much smaller increase was observed over the same period (0.32 vs. 9.46%, *P* < 0.05). However, the difference in *Lactobacillus* relative abundance at 12 months between the two groups was not statistically significant (*P* > 0.05) (Fig. 1d).

Clustering analysis using the Dirichlet Multinomial Mixtures method at 12 months identified four distinct microbiome clusters. Two clusters (dominated by *Lactobacillus*) were associated with higher treatment success rates (71.4 and 80.0%), while the other two clusters (dominated by *Sneathia*, *Prevotella*, *Shuttleworthia*, and *Gardnerella*) had lower success rates (44.4 and 50.0%) (Supplementary Fig. 4).

The alpha diversity was also correlated with HPV status at 12 months. Although no significant difference was observed (*P* > 0.05), HPV-negative women group showed slightly lower alpha diversity than HPV-positive women at 3 and 12 months (Supplementary Fig. 5). Microbial composition analysis revealed that, compared to HPV-negative women, HPV-positive women had higher abundances of pathogens like *Sneathia* (6.1 vs. 11.1%) and *Prevotella* (8.2 vs. 13.2%) and lower levels of *Lactobacillus* (32.7 vs. 41.1%) (Supplementary Fig. 6).

These findings align with previous studies indicating that low diversity of the microbiome, mainly composed of *Lactobacillus* spp., is characteristic of a healthy cervicovaginal environment (Reviewed in [15] and [12–14,21]). The emergence of certain bacteria mainly anaerobic leads to vaginal dysbiosis and may contribute to different diseases [22]. Furthermore, these findings are consistent with studies identifying *Prevotella*, *Gardnerella*, *Sneathia*, and *Fastidiosipila* being specifically associated with HPV infection, while *Porphyromonas* and *Shuttleworthia* were identified as a potential marker of cervical lesions (Reviewed in [23] and [24,25]).

HIV viral load analysis was performed using qPCR (HIV-1 DNA Test PRO, Diatheva, IT) on 84 cervicovaginal cell pellets collected at baseline and after 12 months. Twenty-four samples failed HIV quantification due to unsuccessful amplification of the internal control hTERT gene, likely resulting from DNA fragmentation, degradation, or the presence of PCR inhibitors. At 12 months, the virus was undetectable in all tested women, except for one (<5 copies), consistent with all participants being on antiretroviral therapy (ART). These findings are reassuring, as concerns had been raised about increased HIV viral shedding following cervical precancer treatment due to inflammation and mucosal damage [26,27].

The study had limitations, including the small sample size, the inability to identify species-level microbiome composition due to partial 16S rRNA sequencing, and the absence of a comparator group of women without HIV.

In conclusion, the findings suggest a beneficial role for *Lactobacillus* in improving cervical lesion treatment outcomes. A vaginal microbiome profile dominated by *Lactobacillus* may serve as a potential biomarker for positive treatment responses. Therapeutic approaches, such as probiotics, could help regulate the vaginal microbiome, enhance HPV clearance, and promote the regression of cervical precancer lesions. Further large-scale, multicentric studies are urgently needed to address the challenges of cervical precancer treatment among WWH.

## Acknowledgements

The authors are grateful to Ms Nicole Suty for her help with manuscript preparation, and to Ms Marie-Pierre Cros for her technical support.

All data are fully available without restriction along the manuscript and in the supplementary material. All raw data have been deposited to NCBI's SRA under accession PRJNA1138683.

The study was reviewed and approved by the research ethics committees at IARC (IEC 2016-01 approved on 4 March 2016) and UNC (MH/101/23/10/1 approved on 10 July 2017). Each study participant signed a written informed consent. An information leaflet was shared with the participants and the contents were explained by the study coordinator before obtaining consent. The study was also approved by the IARC scientific council internal review board.

The study was funded by the US National Institute of Health (1UH2CA202721-01).

L.F.P., G.P.P., J.Z., P.B., and T.G. contributed to the design of the study. L.F.P., P.Be, V.C., E.L., M.K., R.W., A.G., Z.H., J.Z., P.B., and T.G. contributed to the data analysis and/or writing of the article. G.P.P., L.F.P., M.M., N.N., S.C., and A.L.S. organized and/or collected the samples in the field. C.C., S.M.C., V.F. performed the DNA extraction of the samples, and/or 16S rRNA sequencing library preparation. All authors read and approved the final manuscript.

### Conflicts of interest

The authors declare that they have no competing interests.

Where authors are identified as personnel of the International Agency for Research on Cancer/WHO, the authors alone are responsible for the views expressed in this article and they do not necessarily represent the decisions, policy, or views of the International Agency for Research on Cancer/WHO.

## Supplementary Material
