# Supplementary material for: Vaginal microbiome composition in women with HIV undergoing treatment of cervical transformation zone in a screen and treat program in Zambia
Source: AIDS. 2025 Jun 26;39(9):1303–6. doi: 10.1097/QAD.0000000000004187 (PMC12204225; doi:10.1097/QAD.0000000000004187)
Supplement: Supplementary file 1 [file aids-39-1303-s001.pptx]

## Slide 1
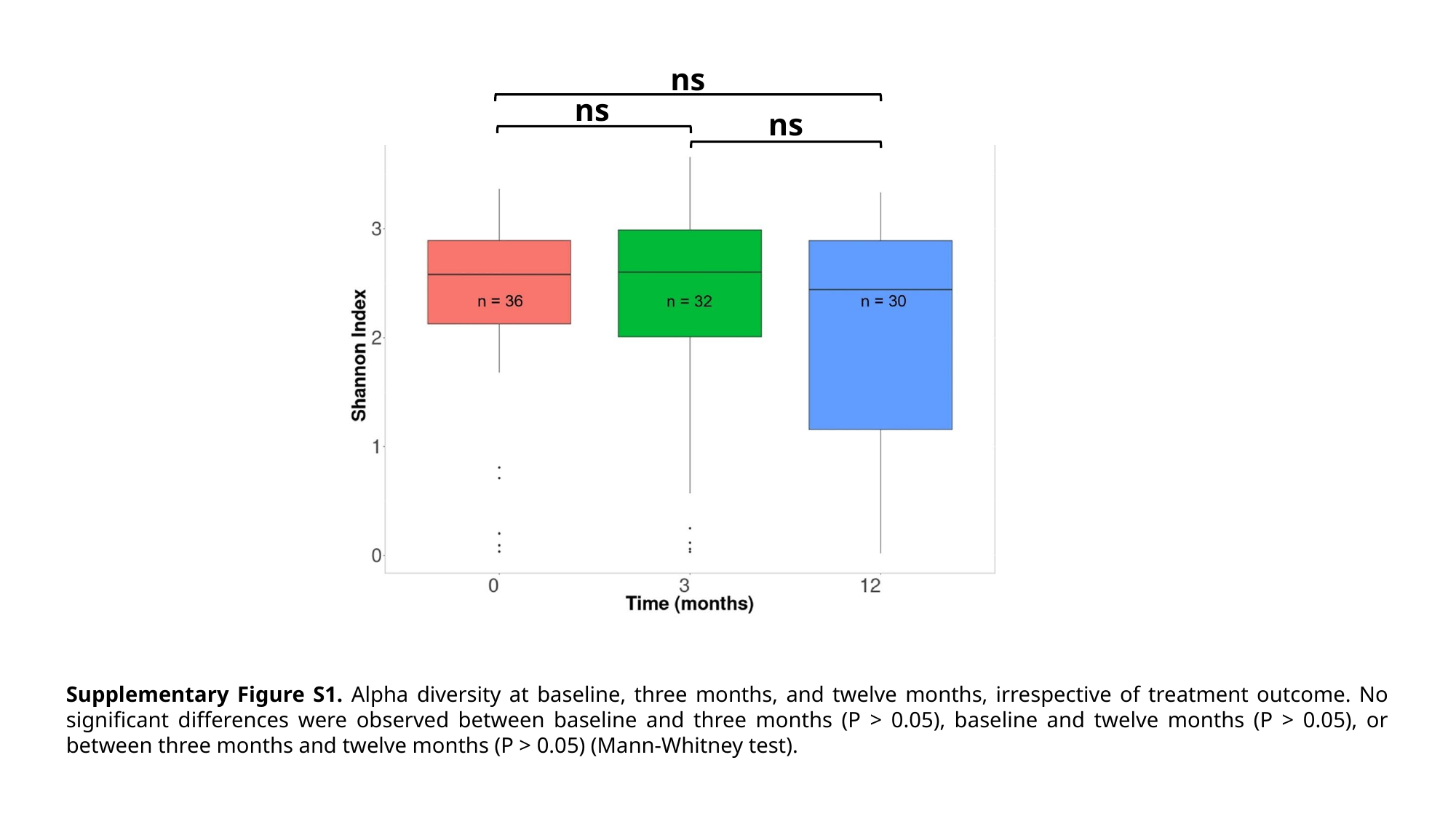

ns
ns
ns
Supplementary Figure S1. Alpha diversity at baseline, three months, and twelve months, irrespective of treatment outcome. No significant differences were observed between baseline and three months (P > 0.05), baseline and twelve months (P > 0.05), or between three months and twelve months (P > 0.05) (Mann-Whitney test).
