# Supplementary material for: Vaginal microbiome composition in women with HIV undergoing treatment of cervical transformation zone in a screen and treat program in Zambia
Source: AIDS. 2025 Jun 26;39(9):1303–6. doi: 10.1097/QAD.0000000000004187 (PMC12204225; doi:10.1097/QAD.0000000000004187)
Supplement: Supplementary file 2 [file aids-39-1303-s002.pptx]

## Slide 1
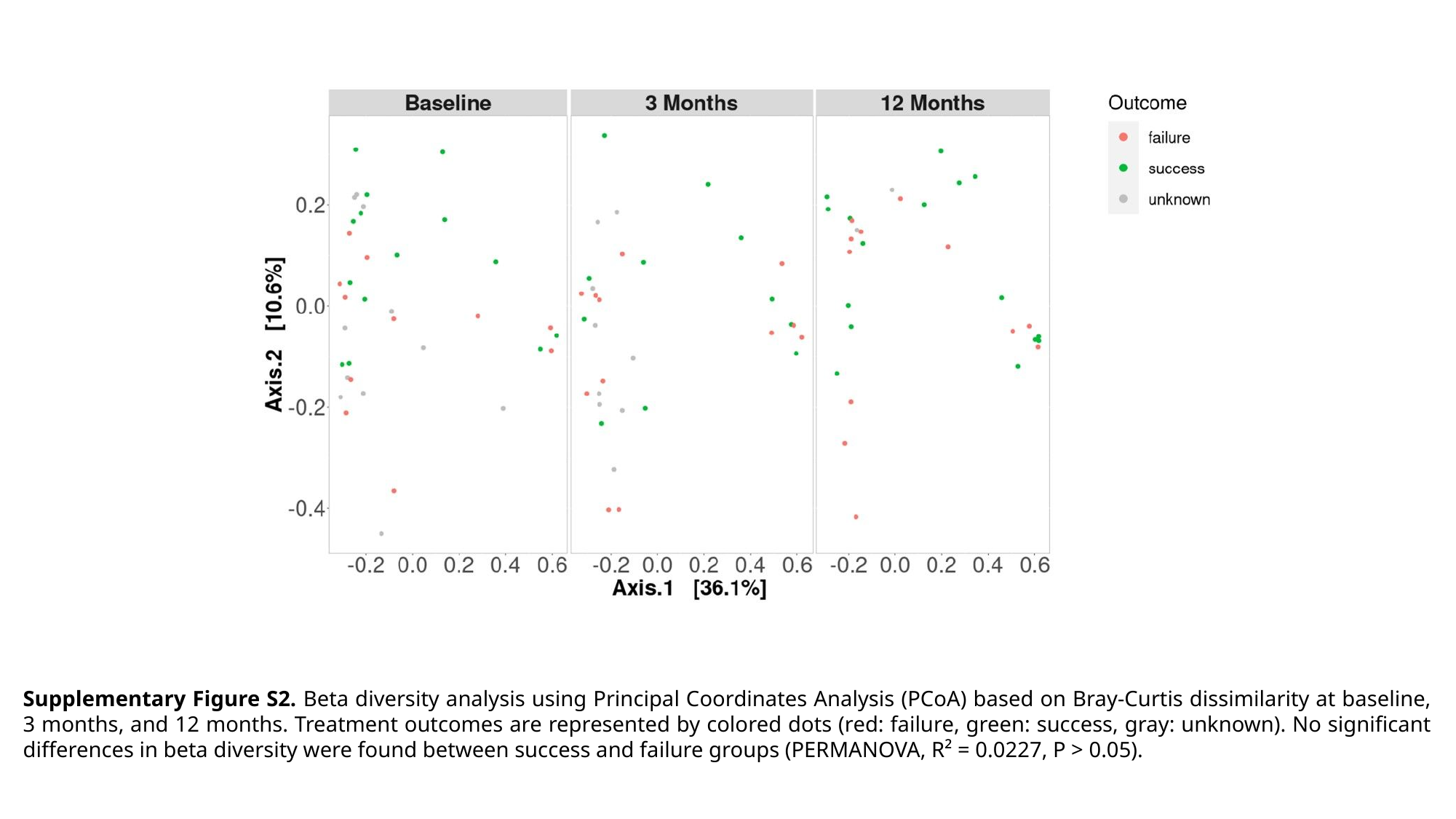

Supplementary Figure S2. Beta diversity analysis using Principal Coordinates Analysis (PCoA) based on Bray-Curtis dissimilarity at baseline, 3 months, and 12 months. Treatment outcomes are represented by colored dots (red: failure, green: success, gray: unknown). No significant differences in beta diversity were found between success and failure groups (PERMANOVA, R² = 0.0227, P > 0.05).
