# Supplementary material for: Vaginal microbiome composition in women with HIV undergoing treatment of cervical transformation zone in a screen and treat program in Zambia
Source: AIDS. 2025 Jun 26;39(9):1303–6. doi: 10.1097/QAD.0000000000004187 (PMC12204225; doi:10.1097/QAD.0000000000004187)
Supplement: Supplementary file 3 [file aids-39-1303-s003.pptx]

## Slide 1
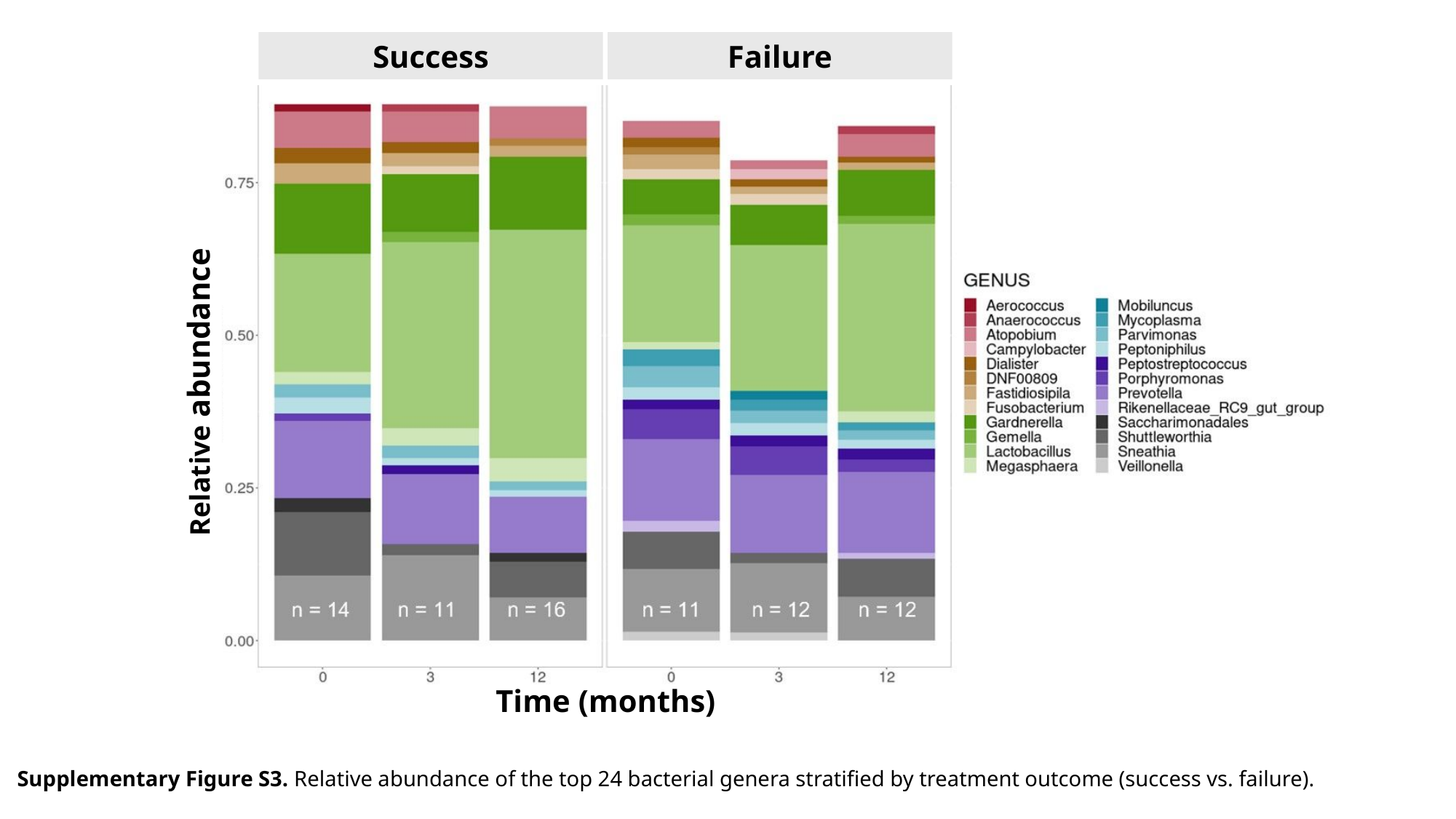

Success
Failure
Relative abundance
Time (months)
Supplementary Figure S3. Relative abundance of the top 24 bacterial genera stratified by treatment outcome (success vs. failure).
