# Supplementary material for: Vaginal microbiome composition in women with HIV undergoing treatment of cervical transformation zone in a screen and treat program in Zambia
Source: AIDS. 2025 Jun 26;39(9):1303–6. doi: 10.1097/QAD.0000000000004187 (PMC12204225; doi:10.1097/QAD.0000000000004187)
Supplement: Supplementary file 4 [file aids-39-1303-s004.pptx]

## Slide 1
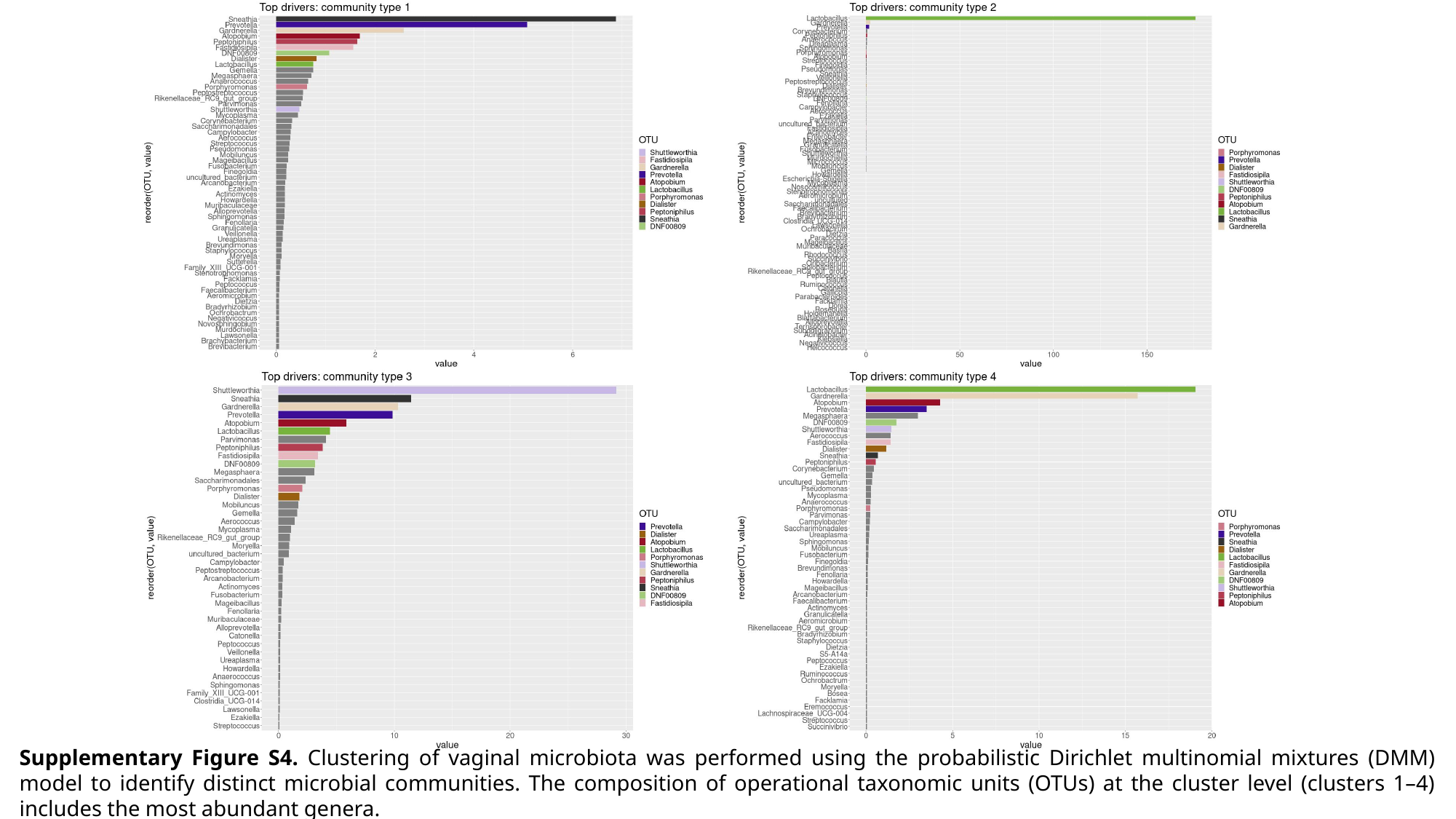

Supplementary Figure S4. Clustering of vaginal microbiota was performed using the probabilistic Dirichlet multinomial mixtures (DMM) model to identify distinct microbial communities. The composition of operational taxonomic units (OTUs) at the cluster level (clusters 1–4) includes the most abundant genera.
