# Supplementary material for: Vaginal microbiome composition in women with HIV undergoing treatment of cervical transformation zone in a screen and treat program in Zambia
Source: AIDS. 2025 Jun 26;39(9):1303–6. doi: 10.1097/QAD.0000000000004187 (PMC12204225; doi:10.1097/QAD.0000000000004187)
Supplement: Supplementary file 5 [file aids-39-1303-s005.pptx]

## Slide 1
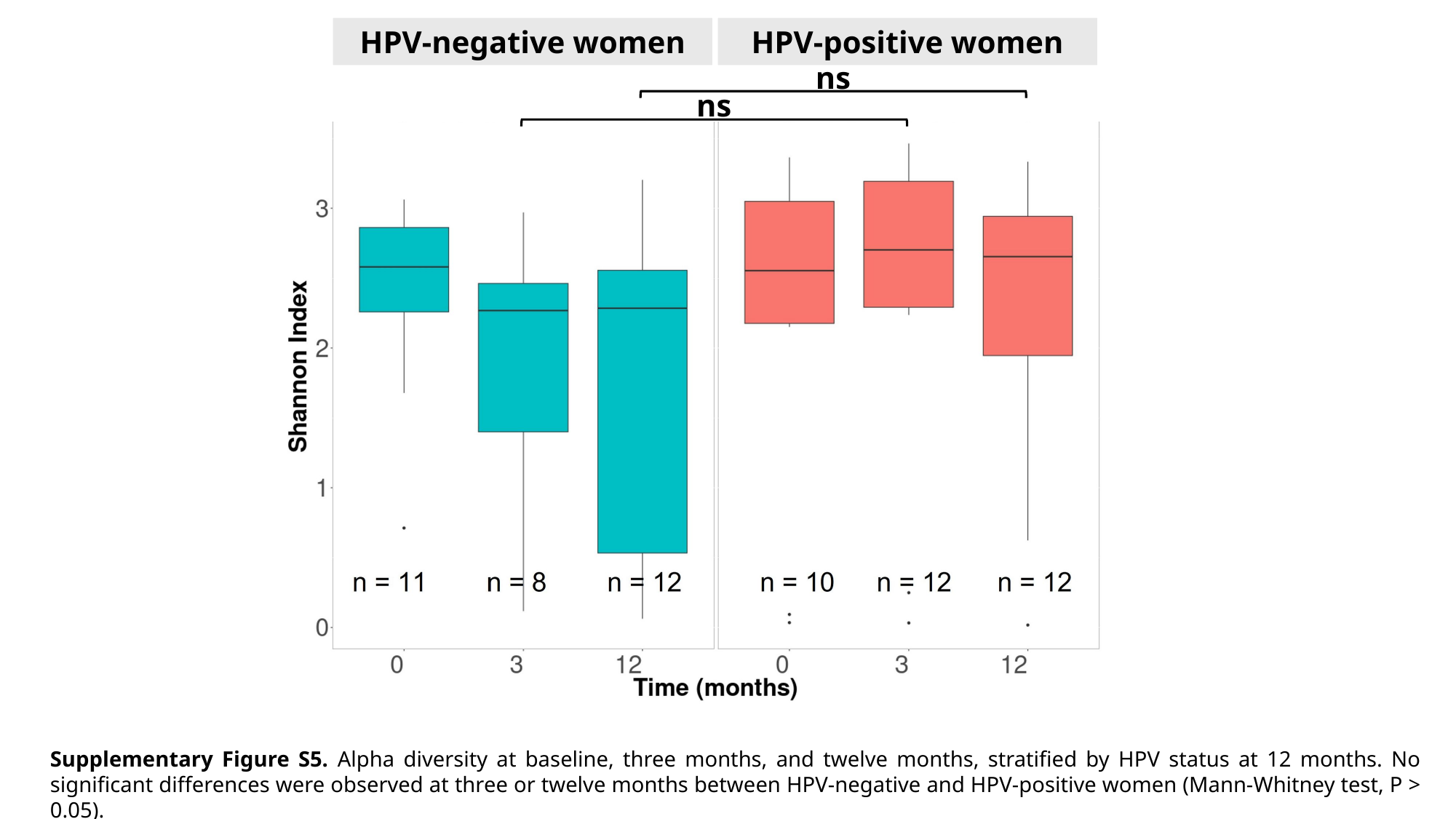

HPV-negative women
HPV-positive women
ns
ns
Supplementary Figure S5. Alpha diversity at baseline, three months, and twelve months, stratified by HPV status at 12 months. No significant differences were observed at three or twelve months between HPV-negative and HPV-positive women (Mann-Whitney test, P > 0.05).
