# Supplementary material for: Vaginal microbiome composition in women with HIV undergoing treatment of cervical transformation zone in a screen and treat program in Zambia
Source: AIDS. 2025 Jun 26;39(9):1303–6. doi: 10.1097/QAD.0000000000004187 (PMC12204225; doi:10.1097/QAD.0000000000004187)
Supplement: Supplementary file 6 [file aids-39-1303-s006.pptx]

## Slide 1
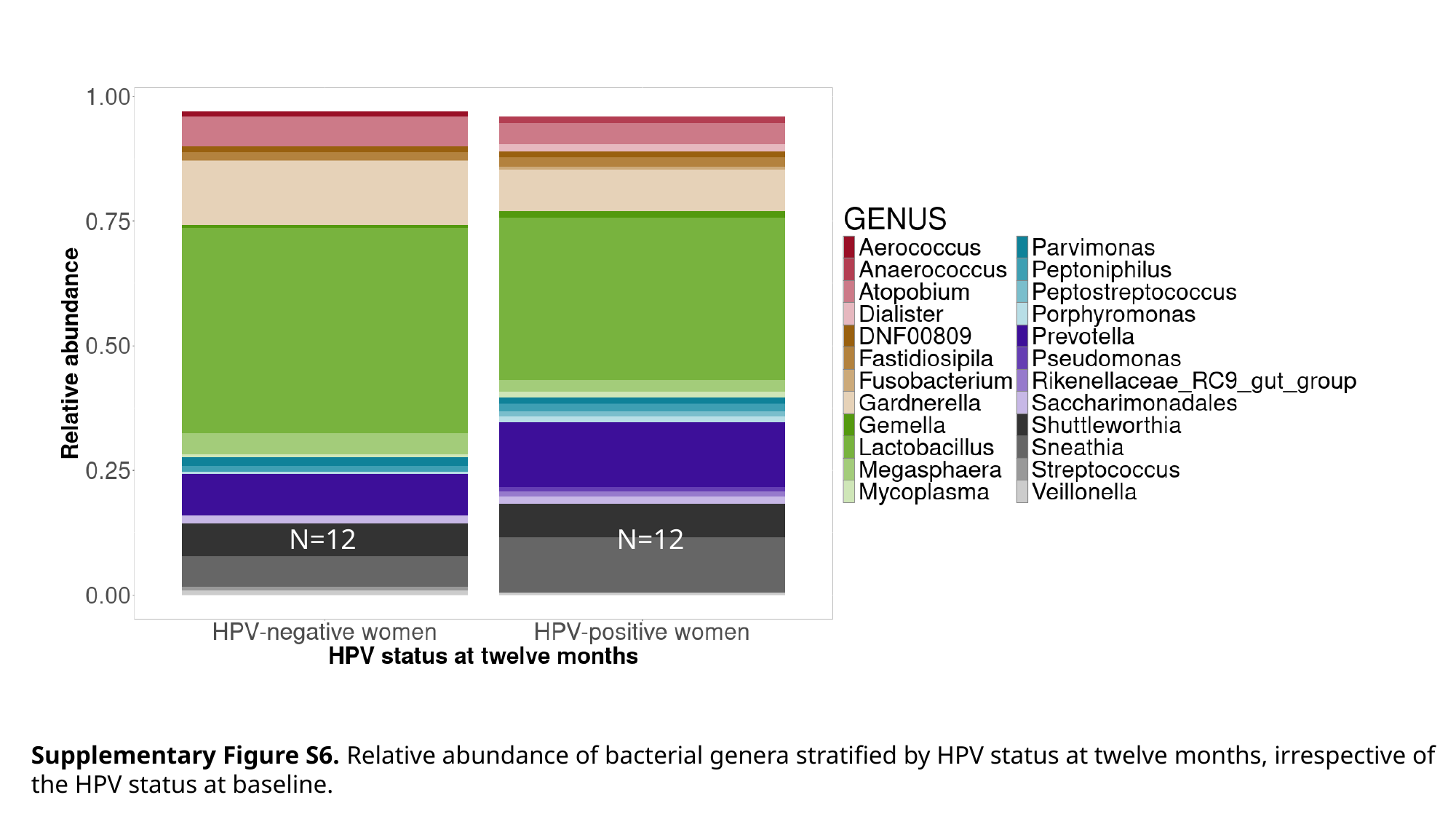

N=12
N=12
Supplementary Figure S6. Relative abundance of bacterial genera stratified by HPV status at twelve months, irrespective of the HPV status at baseline.
