# Supplementary material for: Vaginal microbiome composition in women with HIV undergoing treatment of cervical transformation zone in a screen and treat program in Zambia
Source: AIDS. 2025 Jun 26;39(9):1303–6. doi: 10.1097/QAD.0000000000004187 (PMC12204225; doi:10.1097/QAD.0000000000004187)
Supplement: Supplementary file 7 [file aids-39-1303-s007.docx]

**Supplementary Table S1: Baseline socio demographic characteristics, reproductive health history and clinical details**

|  | **Treatment outcome** | | | | | |  | |  |
| --- | --- | --- | --- | --- | --- | --- | --- | --- | --- |
|  | **Treatment** | | **Treatment** | | **Outcome not** | | **Total** | | **Chi^2^** |
|  | **failure** | | **success^a^** | | **assessable** | |  | |  |
|  | **n (%)** | | **n (%)** | | **n (%)** | | **n (%)** | | **p-value** |
| Participants recruited | 17 |  | 18 |  | 11 |  | 46 |  |  |
| Age (years) |  |  |  |  |  |  |  |  |  |
| 25-39 | 10 | (58.8) | 15 | (83.3) | 10 | (90.9) | 35 | (76.1) | 0.10 |
| 40-59 | 7 | (41.2) | 3 | (16.7) | 1 | (9.1) | 11 | (23.9) |  |
| Participant education |  |  |  |  |  |  |  |  |  |
| None and primary | 9 | (52.9) | 10 | (55.6) | 4 | (36.4) | 23 | (50.0) | 0.58 |
| Secondary and above | 8 | (47.1) | 8 | (44.4) | 7 | (63.6) | 23 | (50.0) |  |
| Occupation |  |  |  |  |  |  |  |  |  |
| Business owners and professional | 9 | (52.9) | 9 | (50.0) | 8 | (72.7) | 26 | (56.5) | 0.45 |
| Others | 8 | (47.1) | 9 | (50.0) | 3 | (27.3) | 20 | (43.5) |  |
| Marital status |  |  |  |  |  |  |  |  |  |
| Currently married/cohabiting | 6 | (35.3) | 13 | (72.2) | 9 | (81.8) | 28 | (60.9) | 0.02 |
| Not currently married/cohabiting | 11 | (64.7) | 5 | (27.8) | 2 | (18.2) | 18 | (39.1) |  |
| Residence area |  |  |  |  |  |  |  |  |  |
| Urban | 13 | (76.5) | 12 | (66.7) | 8 | (72.7) | 33 | (71.7) | 0.81 |
| Semiurban | 4 | (23.5) | 6 | (33.3) | 3 | (27.3) | 13 | (28.3) |  |
| Total pregnancies |  |  |  |  |  |  |  |  |  |
| 0-2 | 8 | (47.1) | 9 | (50.0) | 3 | (27.3) | 20 | (43.5) | 0.45 |
| 3+ | 9 | (52.9) | 9 | (50.0) | 8 | (72.7) | 26 | (56.5) |  |
| Total number of live births |  |  |  |  |  |  |  |  |  |
| 0-2 | 9 | (52.9) | 10 | (55.6) | 7 | (63.6) | 26 | (56.5) | 0.85 |
| 3+ | 8 | (47.1) | 8 | (44.4) | 4 | (36.4) | 20 | (43.5) |  |
| Last menstruation |  |  |  |  |  |  |  |  |  |
| < 12 months | 13 | (76.5) | 18 | (100.0) | 9 | (81.8) | 40 | (87.0) | 0.10 |
| 12 months+ | 4 | (23.5) | 0 | (0.0) | 2 | (18.2) | 6 | (13.0) |  |
| Duration with HIV infection (years) |  |  |  |  |  |  |  |  |  |
| <2 | 10 | (58.8) | 5 | (27.8) | 5 | (45.5) | 20 | (43.5) | 0.18 |
| 2+ | 7 | (41.2) | 13 | (72.2) | 6 | (54.5) | 26 | (56.5) |  |
| Patient on ART |  |  |  |  |  |  |  |  |  |
| On ART | 17 | (100.0) | 18 | (100.0) | 11 | (100.0) | 46 | (100.0) |  |
| HPV testing results |  |  |  |  |  |  |  |  |  |
| Negative | 3 | (18.8) | 8 | (44.4) | 0 | (0.0) | 11 | (24.4) | 0.02 |
| Positive | 13 | (81.3) | 10 | (55.6) | 11 | (100.0) | 34 | (75.6) |  |
| HPV type |  |  |  |  |  |  |  |  |  |
| HPV 16 | 5 | (38.5) | 2 | (20.0) | 2 | (18.2) | 9 | (26.5) | 0.77 |
| HPV 18 and/or 45 | 3 | (23.1) | 4 | (40.0) | 4 | (36.4) | 11 | (32.4) |  |
| HPV 31, 33, 35, 39, 51, 52, 56, 58, 59, 66 and/or 68 | 5 | (38.5) | 4 | (40.0) | 5 | (45.5) | 14 | (41.2) |  |
| Baseline high risk HPV status |  |  |  |  |  |  |  |  |  |
| HPV negative | 3 | (18.8) | 8 | (44.4) | 0 | (0.0) | 11 | (24.4) | 0.02 |
| HPV positive | 13 | (81.3) | 10 | (55.6) | 11 | (100.0) | 34 | (75.6) |  |
| Month 12 follow-up status |  |  |  |  |  |  |  |  |  |
| Not eligible for follow up (Dead) | 0 |  | 0 |  | 1 |  | 1 |  |  |
| Eligible for follow-up | 17 |  | 18 |  | 10 |  | 45 |  |  |
| Noncompliant to follow up | 0 |  | 0 |  | 10 |  | 10 |  |  |
| Followed up | 17 | (100.0) | 18 | (100.0) | 0 | (0.0) | 35 | (77.8) |  |
| Lesion persistence type |  |  |  |  |  |  |  |  |  |
| HPV 16 persistence | 3 | (17.6) |  |  |  |  |  |  |  |
| HPV 18 and/or 45 persistence | 3 | (17.6) |  |  |  |  |  |  |  |
| Persistence of other HPV types | 7 | (41.2) |  |  |  |  |  |  |  |
| VIA positivity persistence | 4 | (23.5) |  |  |  |  |  |  |  |
| LLETZ: Large loop excision of the transformation zone; HIV: human immunodeficiency virus; ART: antiretroviral therapy; HPV: human papilloma virus  ^a^No evidence of disease was defined as either a) HPV clearance during follow-up of the same HPV type detected at baseline or b) a VIA-negative finding at follow-up among women who were HPV-negative at baseline. Participants with an unknown follow-up outcome status were those for whom no follow-up information was available. Of note, Follow-up VIA findings were used among women with baseline HPV negative results since follow-up HPV testing was done only among women with baseline HPV positive results as was stated in the protocol. | | | | | | | | | |
